# Intelligent Detection and Description of Foreign Object Debris on Airport Pavements via Enhanced YOLOv7 and GPT-Based Prompt Engineering

**DOI:** 10.3390/s25165116

**Published:** 2025-08-18

**Authors:** Hanglin Cheng, Ruoxi Zhang, Ruiheng Zhang, Yihao Li, Yang Lei, Weiguang Zhang

**Affiliations:** 1 School of Transportation, Southeast University, Nanjing 211189, China; 2 Luoyang Flight College, Civil Aviation Flight University of China, Luoyang 471132, China

**Keywords:** foreign object debris, small-object detection, segment anything, structured prompt engineering

## Abstract

Foreign Object Debris (FOD) on airport pavements poses a serious threat to aviation safety, making accurate detection and interpretable scene understanding crucial for operational risk management. This paper presents an integrated multi-modal framework that combines an enhanced YOLOv7-X detector, a cascaded YOLO-SAM segmentation module, and a structured prompt engineering mechanism to generate detailed semantic descriptions of detected FOD. Detection performance is improved through the integration of Coordinate Attention, Spatial–Depth Conversion (SPD-Conv), and a Gaussian Similarity IoU (GSIoU) loss, leading to a 3.9% gain in mAP@0.5 for small objects with only a 1.7% increase in inference latency. The YOLO-SAM cascade leverages high-quality masks to guide structured prompt generation, which incorporates spatial encoding, material attributes, and operational risk cues, resulting in a substantial improvement in description accuracy from 76.0% to 91.3%. Extensive experiments on a dataset of 12,000 real airport images demonstrate competitive detection and segmentation performance compared to recent CNN- and transformer-based baselines while achieving robust semantic generalization in challenging scenarios, such as complete darkness, low-light, high-glare nighttime conditions, and rainy weather. A runtime breakdown shows that the enhanced YOLOv7-X requires 40.2 ms per image, SAM segmentation takes 142.5 ms, structured prompt construction adds 23.5 ms, and BLIP-2 description generation requires 178.6 ms, resulting in an end-to-end latency of 384.8 ms per image. Although this does not meet strict real-time video requirements, it is suitable for semi-real-time or edge-assisted asynchronous deployment, where detection robustness and semantic interpretability are prioritized over ultra-low latency. The proposed framework offers a practical, deployable solution for airport FOD monitoring, combining high-precision detection with context-aware description generation to support intelligent runway inspection and maintenance decision-making.

## 1. Introduction

Foreign Object Debris (FOD) on airport pavements poses a serious threat to aviation safety, as even small debris can cause significant aircraft damage, operational delays, and costly repairs [1,2]. According to international safety statistics, FOD-related incidents have resulted in substantial economic losses and safety hazards worldwide. Existing detection systems primarily focus on object localization without providing comprehensive semantic understanding, which limits their usefulness in supporting real-time decision-making for runway maintenance and safety management. Moreover, these systems often struggle under challenging environmental conditions, such as low visibility, glare, or adverse weather, reducing their reliability in practical deployment. With modern airports facing escalating operational complexity and increased surface traffic density, conventional inspection methods—such as manual patrols or stationary radar-based solutions—no longer suffice for ensuring continuous, high-resolution, and precise monitoring [3,4,5]. Consequently, there is a pressing demand for intelligent vision-based detection systems capable of addressing persistent challenges, like small-object sizes, ambiguous visual features, cluttered backgrounds, and diverse debris categories [6,7,8,9].

Recent advancements in object detection technologies, especially through the advent of sophisticated frameworks, such as the YOLO series, DETR, and DINO, have shown notable potential in real-time applications [10,11,12,13]. Specifically, techniques involving attention mechanisms, multi-scale feature fusion, and spatial-channel decoupling have considerably enhanced small-object detection capabilities [14,15,16,17]. Furthermore, transformer-based detection architectures introduced cross-scale reasoning and dynamic query refinement to achieve better contextual awareness [18,19]. Despite these advancements, current methodologies continue to encounter limitations in accurately detecting small and weakly textured objects in densely cluttered scenarios, often resulting in localization inaccuracies, anchor box imbalances, and intensive computational demands. These issues critically restrict their deployment in resource-limited edge computing environments common to airports [20,21,22,23].

Recent studies have also sought to improve the detection of small objects through architectural innovations, such as feature pyramid networks (FPNs), recursive feature enhancement, and lightweight attention, which embeds precise positional cues into channel attention maps [24,25,26]. Complementary strategies, like deformable convolutions, have been employed to retain fine-grained spatial information during downsampling. Meanwhile, adaptive label assignment approaches—ranging from Soft-NMS and Distribution Focal Loss to Gaussian similarity-based matching—are increasingly utilized to mitigate positive sample imbalance and enhance the localization stability of detectors, particularly in high-density small-object scenes [27,28,29,30]. However, striking a balance between detection precision, model compactness, and inference speed remains a core challenge, especially for real-time deployments in surveillance-heavy environments, such as runways, taxiways, and apron zones.

In parallel, efforts to improve generalization to unseen categories—such as zero-shot detection and mask-guided segmentation—have advanced significantly, driven by a need to address the inherent limitations of traditional closed-set classification paradigms [31,32]. Foundation models, notably CLIP and the Segment Anything Model (SAM), have demonstrated remarkable generalization capabilities for segmenting previously unseen objects [33,34]. Recent advances in object detection and scene understanding span several methodological paradigms, including CNN-based detectors, transformer-based architectures, diffusion-based generative models for synthetic data augmentation, and foundation models for multi-modal reasoning. However, direct applications of these models to airport-specific FOD scenarios frequently yield coarse segmentation masks lacking precise spatial grounding [35]. To mitigate these shortcomings, recent studies advocate cascaded frameworks integrating object detection and guided segmentation, utilizing detection-generated bounding boxes as prompts to refine mask accuracy. Moreover, interpretability and explainability in intelligent surveillance systems are increasingly emphasized, leading researchers to explore structured prompt engineering, visual–semantic alignment, and context-aware language generation to produce precise and contextually relevant semantic descriptions from large language models [36,37,38,39]. Nevertheless, ensuring robust adaptability across varying scenes, maintaining prompt consistency, and aligning semantic output with visual context remain significant challenges.

In this context, zero-shot segmentation approaches have gained popularity by leveraging vision–language pretraining, used to generate context-aware semantic outputs [40]. Nonetheless, these models often struggle in domain-specific environments, like airport runways, due to limited grounding ability, vague prompt control, and semantic drift in complex scenes [41,42]. To improve segmentation precision and semantic consistency, hybrid approaches have emerged that utilize multi-modal prompts and incorporate spatial priors during mask generation [43]. Simultaneously, structured prompt engineering has evolved from static templates to dynamic, spatially-aware, domain-specific formulations, enabling language models to produce more interpretable and task-aligned descriptions [44,45,46]. Despite these developments, achieving robustness across diverse environmental conditions and maintaining consistency across large-scale deployments remain pressing issues.

**Figure 1:**
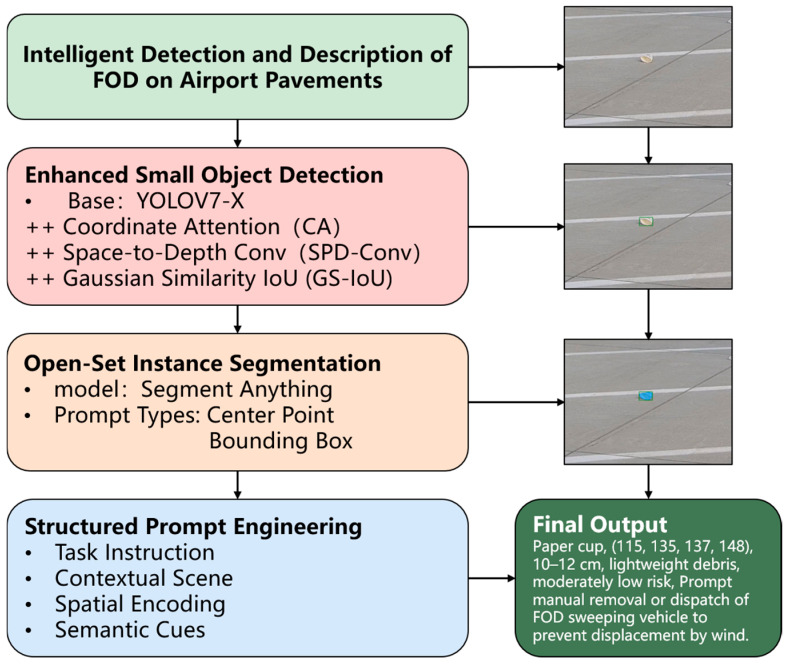
The proposed framework for intelligent FOD detection and semantic interpretation.

To address these limitations, this study proposes a multi-modal YOLO-SAM-GPT framework that integrates enhanced small-object detection, instance-level segmentation, and structured prompt-based semantic description generation, as shown in Figure 1. This combination not only improves detection precision under complex visual conditions but also produces interpretable scene descriptions that convey object category, spatial context, and material attributes, directly supporting operational tasks, such as runway inspection, maintenance planning, and incident reporting. By coupling high detection accuracy with semantic interpretability, the proposed solution enhances the practical applicability of FOD monitoring systems in real-world airport environments, aligning with current trends in intelligent visual perception and safety-critical AI deployment.

## 2. Methodology

### 2.1. Small-Object Detection Optimization

#### 2.1.1. Coordinate Attention Feature Enhancement

**Figure 2:**
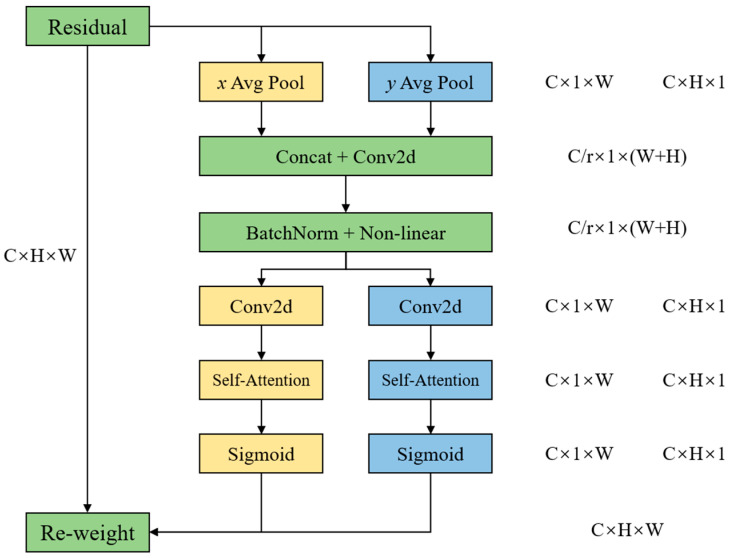
The architecture of the CA module.

The Coordinate Attention (CA) mechanism is designed to enhance small-object detection by embedding spatial location information into channel attention maps. Unlike conventional channel attention, which focuses solely on channel-wise relationships, CA decomposes global pooling into one-dimensional encoding along horizontal and vertical directions, as shown in Figure 2. This enables the model to capture long-range dependencies and precise positional information simultaneously.

Given an input feature map $X\in R^{C\times H\times W}$, Coordinate Attention first applies global average pooling along each axis:

(1) $$z_{c}^{h}\left(i\right)=\frac{1}{W}\sum_{0\leq i\leq W}\;x_{c}\left(i,j\right)$$

(2) $$z_{c}^{w}\left(j\right)=\frac{1}{H}\sum_{0\leq i\leq H}\;x_{c}\left(i,j\right)$$

where $z_{c}^{h}\in R^{C\times H\times 1}$ and $z_{c}^{w}\in R^{C\times 1\times W}$.

These are concatenated and passed through a shared 1 × 1 convolution with activation:

(3) $$f=\delta \left(Conv1\times 1\left(\left[z_{c}^{h},z_{c}^{w}\right]\right)\right)$$

where δ is a non-linear activation. The intermediate representation f is then split and transformed via two separate 1 × 1 convolutions:

(4) $$g^{h}=\sigma (Conv1\times 1(f^{h}))$$

(5) $$g^{w}=\sigma (Conv1\times 1(f^{w}))$$

where σ is the sigmoid function. Finally, the original feature map is re-weighted:

(6) $$Y(i,j)=X(i,j)\cdot g^{h}(i)\cdot g^{w}(j)$$

#### 2.1.2. Space-to-Depth Convolution (SPD-Conv) Module

**Figure 3:**
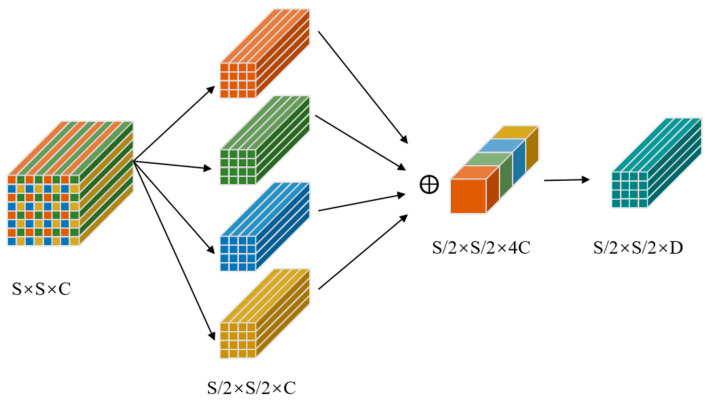
The architecture of the SPD-Conv module.

The SPD-Conv module replaces stride convolutions and pooling to preserve fine-grained features critical for small-object detection, as shown in Figure 3. It first applies a space-to-depth transformation:

Given $\;X\in R^{S\times S\times C}$, it is split into scale^2^ subregions (scale = downsample factor):

(7) $$X^{\prime }=Concat\left(\mathrm{SubRegions}(X)\right),\;X^{\prime }\in R^{\left(S/scale)\times (S/scale)\times (C\cdot {scale}^{2}\right)}$$

This effectively downsamples while retaining all spatial information in channel form. It is followed by a non-strided convolution:

(8) $$Y=Conv(X^{\prime })$$

#### 2.1.3. Gaussian Similarity IoU (GS-IoU) for Bounding Box Approximation

**Figure 4:**
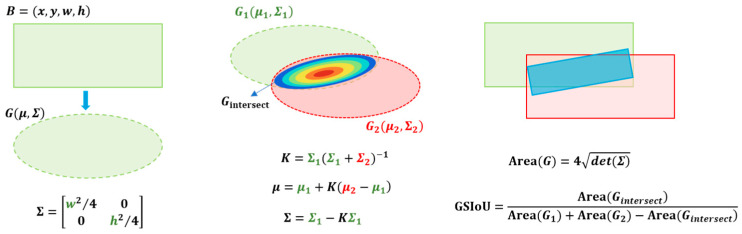
The principle of GS-IoU.

Traditional IoU-based metrics often suffer from instability in scenarios involving small-object detection, especially when slight deviations in predicted box locations lead to zero-overlap penalties. To address this limitation, a Gaussian Similarity IoU (GS-IoU) metric is proposed that approximates non-rotated bounding boxes as 2D Gaussian distributions and estimates spatial overlap probabilistically, as shown in Figure 4.

Given a non-rotated bounding box B=(x,y,w,h), we model it as a 2D Gaussian distribution G(μ,Σ), where the mean is defined as the box center $\mu =(x,y{)}^{T}$, and the covariance matrix Σ is given by

(9) $$\Sigma =\left[\begin{array}{cc} w^{2}/4 & 0\\ 0 & h^{2}/4 \end{array}\right]$$

This transformation effectively converts an axis-aligned box into an elliptical probability distribution with its axes aligned to the coordinate axes.

Given two bounding boxes $B_{1}$ and $B_{2}$, we construct their corresponding Gaussians $G_{1}(\mu_{1},\Sigma_{1})$ and $G_{2}(\mu_{2},\Sigma_{2})$. The probabilistic intersection is approximated by a Kalman Filter-based fusion:

(10) $$K=\Sigma_{1}(\Sigma_{1}+\Sigma_{2}{)}^{-1}$$

(11) $$\mu =\mu_{1}+K(\mu_{2}-\mu_{1})$$

(12) $$\Sigma =\Sigma_{1}-K\Sigma_{1}$$

The resulting Gaussian $G_{intersect}(\mu ,\Sigma )$ serves as a soft estimation of the overlapping region.

To estimate the union region, we construct a union Gaussian distribution $G_{union}$ based on a covariance-weighted averaging scheme. This approach combines the means and covariances of $G_{1}$ and $G_{2}$, ensuring consistency with the Gaussian modeling of the intersection and maintaining analytical differentiability. The resulting union distribution is characterized by a new mean $\mu_{u}$ and covariance matrix $\Sigma_{u}$.

The GS-IoU is then defined as the area ratio:

(13) $$GS-IoU=\frac{Area(G_{intersect})}{Area(G_{1})+Area(G_{2})-Area(G_{intersect})}$$

where $Area(G)=4\sqrt{det(\Sigma )}$, with the area of the 1-standard-deviation ellipse corresponding to the Gaussian.

### 2.2. Mask-Based Semantic Expansion

#### 2.2.1. Cascaded Detection and Prompted Segmentation via YOLO-SAM

To extend the semantic reasoning of FOD beyond predefined categories, we propose a cascaded architecture that integrates discriminative object localization with prompt-driven segmentation. The framework combines a YOLO-based detector with a segmentation module derived from the SAM, wherein bounding box predictions serve as prompts for instance-aware mask generation.

Given an input image I, YOLO outputs a set of bounding boxes $B_{i}$. Each bounding box is mapped to a prompt $P(B_{i})$, which guides the segmentation model to produce a corresponding binary mask $M_{i}$:

(14) $$M_{i}=SAM(I,P\left(B_{i}\right),S)$$

Here, S denotes the scale context encoding introduced to enhance segmentation consistency across FOD of varying sizes. The prompt $P(B_{i})$ is a structured representation that combines both the center point and geometric extent of $B_{i}$, enabling the model to encode both the location and spatial structure. To maintain tight alignment between the predicted masks and bounding boxes, we incorporate a mask consistency loss:

(15) $$L_{mask}=\sum_{i}\;IoU(M_{i},{\hat{B}}_{i})$$

where ${\hat{B}}_{i}$ is the mask-derived bounding region of $M_{i}$, ensuring the segmentation preserves the coarse localization provided by YOLO. Additionally, the SAM is extended to operate over a multi-scale prompt feature hierarchy, where pyramid-level prompts are extracted from different receptive fields, supporting fine-to-coarse matching across scale-diverse FOD objects.

This cascade transforms low-resolution YOLO boxes into spatially precise masks, forming the basis for downstream semantic reasoning and potential generalization to unannotated object types.

#### 2.2.2. Multi-Modal Semantic Prompting with Domain Knowledge

To generate interpretable descriptions of segmented FOD instances, we introduce a multi-modal semantic generation pipeline built upon LLMs, such as GPT. Each object-level description is conditioned on a structured prompt constructed from spatial, contextual, and environmental information. The prompt is defined as

(16) $${Prompt}_{i}=f(C,S_{i},E_{i},D_{i},V_{i})$$

where

C: Task context (“describe runway FOD and assess operational risk”);

$S_{i}$: Spatial encoding of object i (e.g., top-left to bottom-right index);

$E_{i}$: Visual environment (e.g., lighting, surface texture);

$D_{i}$: Domain prior retrieved from an FOD risk ontology;

$V_{i}$: Visual–semantic embedding (e.g., CLIP feature of $M_{i}$).

By embedding $V_{i}=\phi (M_{i},I)$ into the prompt, the model bridges textual and visual semantics, aligning perceptual cues with language generation. The description is then generated via

(17) $${\mathrm{Description}}_{i}=GPT({Prompt}_{i},B_{i})$$

To enhance factual correctness and domain specificity, we integrate a lightweight knowledge graph $G_{FOD}$ that encodes relationships between FOD types, material hazards, and risk levels, enabling the model to inject safety-critical reasoning into the output. More detailed information on the prompt templates, semantic lexicon, and generation usage can be found in Appendix A.

### 2.3. Evaluation Metrics

#### 2.3.1. Evaluation Metrics of Detection Performance

A comprehensive set of metrics is adopted to evaluate the model’s detection performance from multiple perspectives, including accuracy, speed, robustness, and deplorability.

(a) Mean Average Precision (mAP)

The principal metric for detection performance, calculated as the mean of Average Precision (AP) across all classes:

(18) $$mAP=\frac{1}{C}\sum_{i=1}^{C}\;{AP}_{i}$$

where C is the number of classes. In this work, we treat FOD as a single class, so mAP equals AP. AP is derived from the precision–recall curve:

(19) $$Precision=\frac{TP}{TP+FP}$$

(20) $$Recall=\frac{TP}{TP+FN}$$

(b) AP_s_, AP_m_, AP_l_

These represent the AP for small, medium, and large objects, respectively, and help quantify the model’s sensitivity to object scale.

(c) Frames Per Second (FPS)

A key efficiency metric, defined as

(21) $$FPS=\frac{1000}{T_{pre}+T_{infer}+T_{post}}$$

where $T_{pre},T_{infer}$, and $T_{post}$ are the times for preprocessing, inference, and post-processing per image, measured in milliseconds.

#### 2.3.2. Evaluation Metrics for Semantic Description Quality

To comprehensively evaluate the quality of generated environmental descriptions for FOD images, a set of quantitative metrics is introduced that reflects different aspects of semantic alignment, linguistic quality, and computational efficiency.

(a) Description Accuracy

Description accuracy measures the degree of semantic alignment between the generated description and the human-annotated reference, based on key semantic elements. First, a keyword set $K=\{k_{1},k_{2},\ldots ,k_{n}\}$ is constructed from manual annotations of each test image, covering critical scene attributes, such as object category, spatial context, and material properties. Given a generated description, the number of matched keywords is counted as $N_{match}$ and normalized by the total number of reference keywords $N_{ref}$ to obtain the accuracy:

(22) $$Accuracy=\frac{N_{match}}{N_{ref}}$$

A description is considered semantically accurate only if all extracted matches are contextually correct with respect to the annotated scene. This ensures that the metric evaluates the preservation of essential semantic content rather than superficial text similarity. The final accuracy score is averaged over all test samples to provide a dataset-level measure of semantic alignment.

(b) Detail Richness

Detail richness evaluates whether the description includes a sufficient variety of semantic elements, measured by the entropy of keyword distribution.

(23) $$Entropy=-\sum_{i=1}^{n}\;p_{i}logp_{i}$$

where $p_{i}$ is the probability of occurrence of the ith information category.

(c) Language Fluency

Language fluency assesses the naturalness, grammatical correctness, and lexical coherence of the generated sentences using N-gram-based sentence similarity, specifically the BLEU score:

(24) $$BLEU-N=BP\cdot exp\left(\sum_{n=1}^{N}\;w_{n}\log p_{n}\right)$$

where $p_{n}$ is the precision of N-gram matches, $w_{n}$ = 1/N is the weighting factor, and BP is the brevity penalty.

(d) Prompt Controllability

Prompt controllability measures whether the generation aligns with the predefined keywords or guiding prompts. Let K be the number of keywords in the prompt and $K_{hit}$ be the number of those found in the generated description.

(25) $$\mathrm{Prompt}\;\mathrm{Control}\;\mathrm{Rate}=\frac{K_{hit}}{K}$$

(e) Average Inference Time

Average inference time records the time $t_{i}$ taken to generate a description for each image and computes the average over N samples:

(26) $$\mathrm{Average}\;\mathrm{Inference}\;\mathrm{Time}=\frac{1}{N}\sum_{i=1}^{N}\;t_{i}$$

## 3. Experiment

### 3.1. Dataset

**Figure 5:**
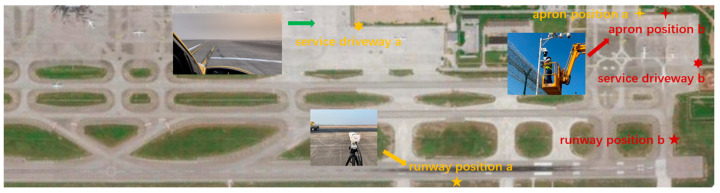
FOD images collection in real airport scenarios.

The dataset used in this study comprises 12,000 annotated images of FOD collected from Luoyang Beijiao Airport and Xuzhou Guanyin Airport, as shown in Figure 5. The images were captured under a wide range of real-world conditions, including daytime, nighttime, rain, fog, and snow, and encompass various zones, such as runway centerlines, taxiways, apron areas, and peripheral zones. The ground surfaces vary from concrete and asphalt to grass and water-covered areas. Each image contains one or more annotated FOD instances, including bounding boxes, masks, semantic labels, and spatial coordinates. Compared to existing open-source FOD datasets, this dataset demonstrates superior diversity, annotation granularity, and environmental realism, offering enhanced generalizability for training and evaluating detection models. To evaluate scale sensitivity, foreign objects are categorized into three groups based on their maximum edge length: small objects (S): max dimension < 32 pixels; medium objects (M): 32 ≤ max dimension < 96 pixels; and large objects (L): max dimension ≥ 96 pixels.

### 3.2. Detection Experiment Details

To rigorously evaluate the detection performance of various models and proposed enhancements, a two-part experimental pipeline was designed: (1) baseline comparison across representative detectors, and (2) ablation studies for module-level improvements. All experiments were conducted on the same computing environment using PyTorch 1.9.0 with CUDA 11.7, and executed on a workstation equipped with an Intel Xeon Gold 6226R CPU, NVIDIA RTX 3090 GPU (24 GB), and 128 GB RAM. All training and evaluation were performed on the proposed airport FOD dataset, which includes 12,000 annotated images under diverse conditions.

For fair comparison, all models were trained under identical hyperparameter settings unless otherwise noted. The initial learning rate was set to 0.01 with an SGD optimizer, momentum = 0.937, and a cosine decay scheduler over 300 epochs. The batch size was fixed at 16, and image resolution was standardized to 640 × 640. Standard data augmentation techniques, including mosaic, random affine, and color jitter, were applied to enhance generalization. For all models, early stopping was employed based on the validation set AP.

The model comparison involved four representative object detectors from the YOLO family: YOLOv5-X, YOLOX-X, YOLOv7, and YOLOv7-X. The goal of this comparison was to explore the trade-off between detection accuracy (AP, AP_s_, AP_m,_ AP_l_) and real-time performance (FPS, model size).

The second part of this study focused on ablation experiments to evaluate the impact of specific architectural modules integrated into YOLOv7-X. The three modules considered are as follows: (a) Coordinate Attention (CA), which enhances location-sensitive channel attention in the detection head; (b) Space-to-Depth Convolution (SPD-Conv), which replaces downsampling layers to preserve local details in early layers; and (c) Gaussian Similarity IoU (GS-IoU), which replaces traditional IoU in label assignment and NMS stages to better accommodate small-object detection with localization uncertainty.

Each module was added incrementally to the base YOLOv7-X model to isolate its individual contribution. All ablation variants were trained with the same configuration as the base model to ensure comparability. For each configuration, we calculate the overall AP and the APs for small, medium, and large objects, alongside changes in FPS and parameter size when relevant.

### 3.3. Open-Set and Incremental Learning

To evaluate the proposed open-set detection and incremental learning strategy under airport scenarios, we designed a multi-stage experiment that combines mask segmentation, semantic reasoning, and continual category expansion. The goal is to enable robust detection of both known and previously unseen FOD categories while also supporting semantic-level interpretation and label evolution over time. The overall framework integrates a YOLO-based object detector, the SAM, and a GPT-based description generator.

In the first stage, the YOLOv7-X detector is trained on a closed-set version of the airport FOD dataset, where 10 base classes are manually defined. These include typical foreign objects, such as plastic bags, screws, nuts, tape, paper, and cloth. Once trained, the YOLO output bounding boxes are used to prompt the SAM. To improve mask quality, the center point and aspect ratio of each bounding box are converted into prompt embeddings, with a filtering threshold applied to exclude boxes with low confidence. The SAM then generates high-resolution segmentation masks corresponding to each object region.

In the second stage, the extracted masks are paired with visual image patches and passed into a GPT-style large language model. This study constructs contextual prompts for foreign object images through two core steps: prompt formulation and application of image captioning models. By integrating these stages, we achieve semantic modeling of the foreign object’s environment and transform it into natural language, providing GPT with high-quality, multi-faceted contextual guidance.

Prompt formulation is based on systematic extraction of environmental elements surrounding the foreign objects, encompassing four semantic categories: ground material, lighting conditions, spatial location, and surrounding facilities. The specific steps are as follows:

(1) Ground material: This is determined via texture analysis combined with CLIP or surface segmentation networks to identify surface types, such as “concrete ground,” “grass area,” “asphalt pavement,” or “water accumulation zone.”
(2) Lighting conditions: These are assessed through image brightness distribution, exposure, and shadow density to describe illumination quality, like “direct sunlight,” “strong shadow interference,” “insufficient brightness,” or “backlit region.”
(3) Spatial location: This is inferred by correlating image boundaries, runway orientation, and airport schematics to locate objects relative to landmarks, e.g., “near runway centerline,” “close to runway edge,” “at apron entrance,” or “along flight path.”
(4) Surrounding facilities: These are identified by object detection or semantic segmentation models to detect adjacent structures, such as “edge lighting strips,” “drainage ditches,” “fences,” or “ground marking lines.”

These semantic elements are combined into standardized prompt templates. For example, “The image shows a foreign object located at the eastern edge of the runway, resting on concrete ground, surrounded by boundary lighting, under sufficient illumination.”

To improve the automation and quality of prompt generation, this study evaluates and selects from leading state-of-the-art image-to-text models, including BLIP-2 (Bootstrapped Language-Image Pretraining), GIT (Generative Image-to-Text Transformer), MiniGPT-4, and PaLI (Pathways Language and Image model). All models were implemented using the PyTorch framework and deployed on a high-performance server equipped with dual NVIDIA A6000 48 GB GPUs, ensuring stable hardware conditions for fair comparison.

## 4. Results and Discussions

### 4.1. Detection Performance Analysis

#### 4.1.1. Backbone Comparison

As shown in Table 1, the AP improves progressively from YOLOv5-X to YOLOv7-X. Compared with YOLOv5-X, YOLOv7-X achieves a 3.4% increase in AP, a 44 FPS gain in inference speed, and a 15.4 MB reduction in model size. In comparison to YOLOX-X, YOLOv7-X achieves 1.3% higher AP, 63 more FPS, and 26.1 MB smaller model size. Relative to YOLOv7, YOLOv7-X adopts a compound scaling strategy, scaling the depth of computation blocks to 1.5 times and the width of transition layers to 1.25 times. This leads to a 1.2% improvement in AP, with small, medium, and large-object APs increased by 0.6%, 1.7%, and 0.3%, respectively. Although the inference speed of YOLOv7-X is 44 FPS lower than the original YOLOv7, its 90 FPS throughput remains sufficient to meet the real-time requirements of airport FOD monitoring. Considering the balance between detection accuracy and inference efficiency, YOLOv7-X is selected as the base detector in this work, upon which further enhancements are developed to improve FOD recognition under complex airport environments.

For completeness, we additionally evaluated YOLOv8-X and Deformable-DETR under the same dataset and training settings [47,48]. YOLOv8-X achieves comparable performance to YOLOv7-X but offers no substantial improvement in small-object APs while having a larger model size and lower FPS, making it less suitable for our real-time deployment constraints. Deformable-DETR demonstrates competitive AP_m_ and AP_l_ but underperforms in AP_s_ and has a significantly larger model size and lower inference speed, which limits its applicability for high-throughput airport surveillance. For these reasons, we focus our subsequent comparisons on the YOLOv7-based variants.

#### 4.1.2. Ablation Study on Small-Object Modules

As shown in Table 2, introducing the SE module resulted in a 0.6% decrease in overall AP, with APs for small and medium objects reduced by 1.7% and 0.2%, respectively, while the AP for large objects slightly increased by 0.2%. The CBAM module improved the overall AP by 1.4%, with gains of 0.3%, 2.1%, and 2.3% for small, medium, and large objects, respectively. The CA module yielded the most significant improvement, boosting the overall AP by 1.9% and improving small, medium, and large-object APs by 0.9%, 2.6%, and 2.7%, respectively. These results indicate that incorporating CA enhances the model’s ability to detect FOD, outperforming both SE and CBAM. Unlike SE and CBAM, CA embeds spatial location information into channel attention, which enhances the extraction of discriminative features, particularly for irregular and small-scale targets.

To evaluate the effectiveness of the SPD-Conv module, we replaced the standard convolution and pooling layers in the shallow backbone with Space-to-Depth Convolution blocks. The comparative results before and after this optimization are shown in Table 3. After incorporating SPD-Conv, the model’s overall AP increased by 2.8%, with APs for small, medium, and large objects improving by 6.0%, 2.4%, and 0.1%, respectively. Notably, the recognition performance for small objects improved significantly. This enhancement is attributed to the fact that, in the original network, deeper layers with larger receptive fields tend to capture high-level semantic features over broader spatial regions, emphasizing coarse object contours. However, small objects contain limited pixel-level information, and the repeated downsampling from convolution and pooling can result in the loss of fine-grained details, leading to missed detections. By applying SPD-Conv in the early layers of the backbone, spatial details are preserved more effectively, thereby improving the model’s ability to detect small-scale targets.

**Figure 6:**
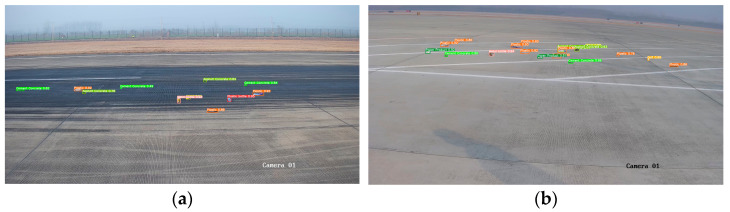
Detection results of FOD using the enhanced YOLO model. (**a**) Runway; (**b**) apron.

To validate the effectiveness of the proposed Gaussian similarity-based label assignment strategy, we conducted experiments using three baseline detectors—Faster R-CNN, YOLOv5-X, and YOLOv7-X—on the airport FOD dataset. As shown in Table 4 and Table 5, incorporating Gaussian similarity into the label assignment process consistently improved detection performance across all models. Specifically, when replacing the traditional IoU-based assignment with Gaussian similarity, the detection performance improved by 5.4%, 5.2%, and 5.8% for Faster R-CNN, YOLOv7-X, and YOLOv8-X, respectively. These results demonstrate that the Gaussian similarity metric more effectively captures spatial correspondence between anchor boxes and the ground truth, especially for small-scale targets, thereby enhancing the overall detection accuracy of anchor-based detectors. Figure 6 illustrates the detection performance of the enhanced YOLO model across distinct airport scenarios, where Figure 6a depicts accurate localization on the runway, and Figure 6b confirms generalization to the apron area.

To assess the efficiency of the proposed GS-IoU, we compared its runtime and training cost with the conventional CIoU on the same hardware setup. As summarized in Table 6, GSIoU reduces the training time per epoch from 68.5 min to 64.7 min, achieving a 5.5% improvement in training efficiency while slightly increasing inference latency by 1.7%. This minor increase in inference time is due to the Gaussian parameterization and similarity computation, but it is outweighed by the performance gains, including a 3.9% improvement in mAP@0.5 for small objects, along with more stable gradient propagation and enhanced localization accuracy.

### 4.2. Semantic Description and Generalization Evaluation

#### 4.2.1. YOLO-SAM Segmentation Results

**Figure 7:**
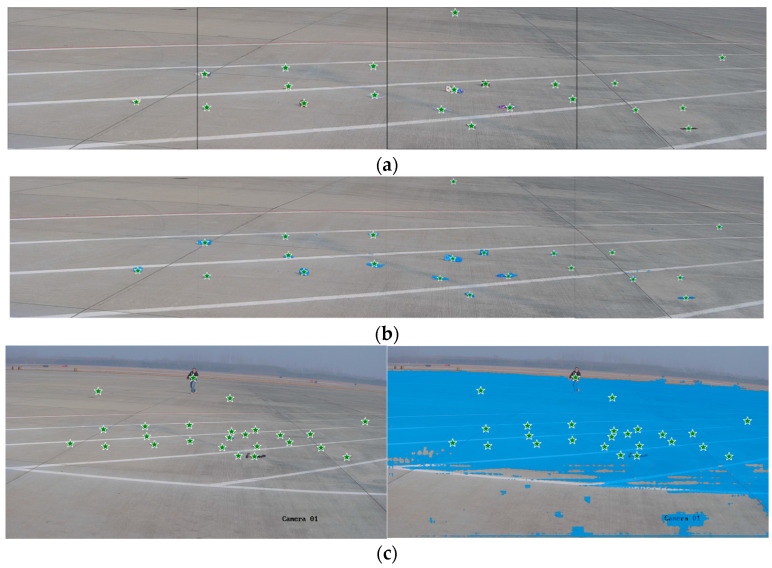
Center point-guided SAM segmentation results. (**a**) YOLO-detected center points of foreign objects; (**b**) segmentation results of the SAM with center point prompts as the input; (**c**) segmentation results of the SAM with multiple center point prompts in a large-scale scene.

Figure 7 presents the segmentation results of the SAM when using YOLO-detected center points as prompt inputs. As shown in Figure 7a, when the image is divided into smaller sub-images, the center points of foreign objects are detected with higher accuracy. Under such conditions, as illustrated in Figure 7b, the SAM successfully segments all foreign objects with well-defined boundaries. However, some runway markings are mistakenly labeled as foreground, indicating partial misclassification. In contrast, as observed in Figure 7c, segmentation performance in large-scale scenes deteriorates despite accurate center point prompts. This decline is primarily due to the high density of foreign objects and the excessive number of prompt points, which result in the generation of a large number of overlapping masks by the SAM. These results suggest that inputting cropped sub-images with reduced object density allows the model to capture more detailed features, thereby improving segmentation accuracy. Therefore, when using center points as prompts for the SAM, it is advisable to preprocess input images by dividing them into appropriately sized sub-images to enhance segmentation precision.

**Figure 8:**
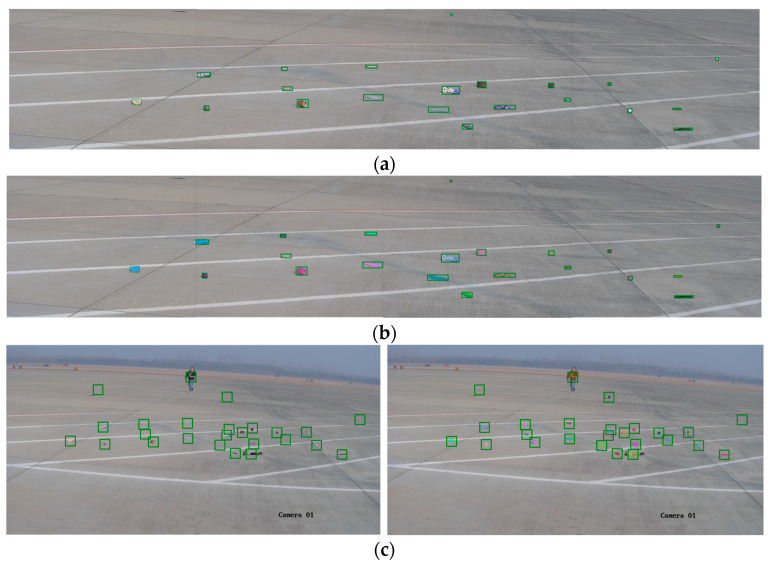
Bounding box-guided SAM segmentation results. (**a**) YOLO-detected bounding boxes of foreign objects; (**b**) segmentation results of the SAM with bounding box prompts as the input; (**c**) segmentation results of the SAM with multiple bounding box prompts in a large-scale scene.

Figure 8 illustrates the segmentation results of the SAM when using YOLO-detected bounding boxes as prompt inputs. As shown in Figure 8a, when the image is divided into smaller patches, the detected bounding boxes of foreign objects are relatively accurate. With precise bounding box prompts, the SAM is able to segment all foreign objects with clear and complete boundaries, as depicted in Figure 8b—yielding better results than center point prompts. When inaccurate bounding boxes are used as inputs, the segmentation performance varies depending on the size of the bounding boxes. As seen in Figure 8c, if the bounding box is too small, the SAM performs segmentation strictly within the box, leaving foreign objects outside the box undetected. However, when a larger bounding box is provided, the model still manages to accurately segment the target. These observations suggest that when the bounding box location is uncertain, applying moderate enlargement can effectively compensate for localization errors and improve segmentation reliability. Therefore, it is recommended to appropriately enlarge the bounding boxes before inputting them into the SAM to ensure accurate segmentation of foreign objects.

Since the output results of the YOLO model do not necessarily correspond to the precise center points and anchor boxes, to further investigate the influence of center point displacement and anchor box scale on the segmentation performance of the SAM, the YOLO-detected results were transformed by applying center point offsets of 0%, 20%, 40%, and 80%, as well as anchor box scaling factors of 50%, 100%, 150%, and 200%. These combinations of offset anchor boxes were then used as the prompts input to the SAM for foreign object segmentation. The segmentation performance of the SAM under different combinations was quantitatively evaluated using MPA, MIoU, and F1 Score metrics. The results are summarized in Table 7.

When the center point offset rate is 0%, anchor box scaling at 100% and 150% yields high segmentation performance with MPA of 96.85, MIoU of 0.982, and F1 Score of 0.983, showing nearly identical results for both scales. However, at 50% scaling, MPA sharply drops to 36.85, MIoU to 0.583, and F1 Score to 0.602, likely due to smaller anchor boxes failing to adequately cover the target under accurate center positioning.

With a 20% center offset, performance declines moderately (MPA 85.24, MIoU 0.875, F1 Score 0.884), while a 150% anchor scaling achieves relatively better results (MPA 88.35, MIoU 0.912, F1 Score 0.932), indicating that increasing anchor size partially compensates for minor center deviations.

At 40% offset, all metrics decrease further, yet 150% scaling still performs best (MPA 82.63, MIoU 0.852, F1 Score 0.865), suggesting larger anchors better adapt to moderate displacement. When offset reaches 80%, performance deteriorates significantly, with the smallest scale (50%) yielding the poorest results (MPA 23.75, MIoU 0.435, F1 Score 0.495). In contrast, 200% scaling improves metrics (MPA 81.84, MIoU 0.832, F1 Score 0.854), as larger anchors more effectively encompass targets despite large offsets.

Overall, segmentation performance declines as the center point offset increases. However, appropriately increasing the anchor box scale can mitigate the negative impact of center displacement. Given the uncertainty of center point accuracy in practice, enlarging YOLO-detected anchor boxes to 150% is recommended as the input prompts to the SAM to enhance segmentation performance.

#### 4.2.2. GPT-Based Description Accuracy

The image dataset used in this study originates from the airport’s foreign object detection system’s operational image repository. To ensure realistic applicability and representativeness, 500 images were selected as the test set, each containing 1 to 5 foreign objects of varying categories with their environmental context fully preserved. These images cover diverse typical airport scenarios, such as runway edges, taxiway intersections, and aprons.

Each image is accompanied by manually annotated environmental descriptions, prepared following the Civil Airport Operation Safety Management Regulations (CCAR-140) and the Foreign Object Debris Prevention Management Measures for Transport Airports (AP-140-CA-2022-05). The annotations include information on object location, ground material, lighting conditions, and adjacent facilities, serving as reference standards for subsequent accuracy evaluation.

For prompt setting, a standardized base prompt—“Please describe the environmental information in the image, including ground material, lighting conditions, spatial location, and surrounding facilities”—was used to ensure prompt controllability and evaluation consistency, testing the models’ responsiveness to semantic cues.

Regarding the generation strategy, one complete natural language description was generated per image and compared semantically to its corresponding manual reference. Five evaluation metrics were calculated: description accuracy, detail richness, language fluency, prompt controllability, and average inference time, as shown in Table 8.

The experiments demonstrate that BLIP-2 achieves the best overall performance, excelling in description accuracy and prompt controllability. This indicates its strong capability to accurately comprehend image environmental semantics while flexibly generating target content based on given prompts. BLIP-2 also maintains high language fluency and moderate inference time, making it well suited for high-precision semantic generation tasks. GIT performs best in language fluency and detail richness, producing naturally structured and information-rich texts, which is advantageous for description tasks emphasizing expressive quality, although its inference speed is relatively slower. MiniGPT-4 leads in inference efficiency at 0.9 s per image, making it suitable for applications requiring real-time performance, but its weaker prompt controllability can cause generated content to deviate from the intended context. PaLI shows moderate performance across metrics, balancing language expression and environmental understanding, and is appropriate for deployment in multitask and multilingual scenarios. Table 9 details the descriptive performance of these four models on foreign object images across varying environments. BLIP-2′s superior accuracy and controllability make it the preferred model for this application, while other models may be selected based on specific trade-offs between performance and efficiency.

#### 4.2.3. Prompt Engineering Ablation

In airport operational environments, foreign objects are typically scattered across runways, taxiways, or adjacent areas with spatial distributions that are inherently uncertain. Without clear ordering and positional encoding, language models may suffer from referential ambiguity, redundant descriptions, or omission errors. Effectively organizing the spatial location information of each detected object is thus a crucial step for generating accurate descriptions and enabling interactive reasoning in multi-object detection and semantic generation tasks. To address this, we propose a spatially ordered foreign object numbering and positional encoding strategy designed to enhance the model’s structural awareness of multiple targets within an image, thereby improving the systematicity and logical clarity of the generated descriptions.

This strategy is based on human visual reading habits, emulating a left-to-right, top-to-bottom ordering to assign unique identifiers to each detected foreign object. Such ordering facilitates the generation of spatially oriented natural language descriptions, such as “the first foreign object,” “the fragment in the upper left corner,” or “the metal object at the far right.” The specific steps include the following:

(a) Mask bounding box extraction: From the mask results produced by the YOLO-SAM, extract the bounding rectangle for each object, obtaining its upper-left $\left(x_{1},y_{1}\right)$ and lower-right $\left(x_{2},y_{2}\right)$ pixel coordinates.
(b) Ordering rule: Sort all detected objects primarily by their $x_{1}$ (horizontal) coordinate and secondarily by their $y_{1}$ (vertical) coordinate, achieving a left-to-right, top-to-bottom sequence and assigning a unique label $\left[i,x_{1},y_{1},x_{2},y_{2}\right]$.
(c) Position normalization: Normalize pixel coordinates relative to image dimensions as x*=x/W, y*=y/H, ensuring spatial information consistency across varying image sizes to facilitate model learning and cross-sample alignment.
(d) Data structure generation: Each foreign object in the image is represented by a triplet consisting of its identifier, spatial location (normalized bounding box), and mask region (image crop or semantic segmentation map). Together, these form a structured spatial prompt accessible to language models.

**Figure 9:**
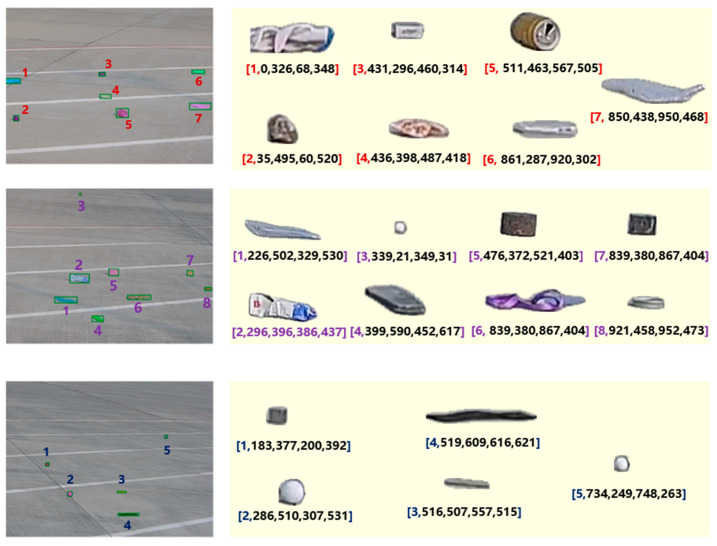
Foreign object mask numbering and spatial localization prompt.

Figure 9 presents detection results for three sample images. The left side shows the numbering and spatial annotations of foreign objects within the original images, while the right side displays the cropped images of each object along with their corresponding spatial encoding information. As illustrated, the model successfully extracts the boundary information of each target and assigns consistent identifiers following the left-to-right, top-to-bottom numbering rule. This provides clear semantic guidance for subsequent multi-object descriptions.

After completing the basic environmental description and spatial information encoding of foreign objects, this study further designs a knowledge-integrated prompt engineering module tailored for the airport domain. The goal is to guide large LLMs to generate more professional, precise, and operationally instructive semantic descriptions of foreign objects through carefully designed natural language instructions. This approach comprises three components: a modular structured prompt design, the construction of a semantic cue lexicon and alignment mechanism, and a prompt generation workflow with model interface integration, forming a closed-loop system from knowledge acquisition, prompt construction, to language generation.

Firstly, to meet the controllability requirements of semantic organization and generation objectives, a four-layer nested structured prompt template is proposed, consisting of four core modules: “Task Instruction,” “Contextual Scene,” “Spatial Encoding,” and “semantic cues”. The Task Instruction explicitly defines the generation target, e.g., “Please describe foreign object number N.” The Contextual Scene module extracts overall scene information from images using multi-modal vision–language models, such as BLIP-2, including ground material, lighting conditions, and area identification. Spatial Encoding combines YOLO-generated bounding box coordinates and object mask maps to achieve precise localization within the image. Semantic cues embed airport foreign object prior knowledge tags, such as typical shapes, material types, and potential source paths. This modular prompt design enhances contextual completeness during language generation and supports rapid prompt reconfiguration for different task types, offering strong generalizability and adaptability.

At the knowledge supply layer, a multi-dimensional semantic cue lexicon is constructed to serve as the foundational resource for the semantic cues module. This lexicon integrates airport operational and maintenance regulations, a historical FOD case database, and expert knowledge from airport operations, organizing keywords into multi-level, multi-dimensional semantic structures. Specifically, it includes 50 typical foreign object categories (e.g., “screw,” “cable head,” “fabric piece”), 12 material attributes (e.g., “metal,” “plastic,” “textile”), 8 spatial risk zones (e.g., “runway centerline area,” “taxiway edge zone”), and 6 maintenance recommendation tags (e.g., “immediate removal required,” “risk controllable”).

In the prompt generation and model application stage, a template-engine-based prompt generation system is developed to automatically assemble structured prompt content according to the visual parsing results of input images, generating natural language instructions compliant with the language model’s input format. The prompts adopt an “instruction-driven + knowledge-constrained” paradigm and are input into GPT-series large language models. Together with image content, object numbering, and mask data, they form a multi-modal input that enables semantic reasoning and accurate description of foreign object scenes.

Comparative experiments were conducted to evaluate the practical effectiveness of the proposed structured prompt engineering framework, covering baseline model performance, module ablation effects, and the impact of semantic enhancement on language generation quality. The experimental setup includes three control groups: Group A uses a baseline model with generic natural language prompts without guidance; Group B introduces spatial location information by incorporating YOLO bounding box coordinates and mask maps as structured inputs; and Group C further integrates the multi-dimensional semantic cue lexicon developed herein, realizing a full-structured prompt input strategy. All groups employ the same language model architecture fine-tuned under identical image and object numbering inputs to ensure comparability.

Table 10 presents the quantitative results of three experimental groups on two key metrics: description accuracy and prompt consistency. The results demonstrate that incorporating spatial localization information increases the description accuracy from 76.4% to 83.7% and improves prompt consistency to 74.8%, indicating that explicit target positioning plays a crucial contextual guidance role during language generation. Furthermore, when combined with the knowledge cue module, Group C achieves a description accuracy of 91.3% and prompt consistency of 89.6%, representing improvements of 14.9 and 28.4 percentage points over the baseline, respectively.

To further investigate the contribution of each semantic element within the structured prompt, we conducted a dimension-specific ablation study. The structured semantic cues used in our prompt design consist of three major dimensions:

S: Spatial location information (e.g., “at the edge of the runway”, “upper-left corner”);

M: Material type (e.g., “metallic debris”, “plastic wrap”);

R: Risk level tag (e.g., “immediate removal required”, “non-critical”).

As shown in Table 11, we observed consistent improvements in both description accuracy and prompt consistency as more semantic dimensions were included. Spatial encoding (S) alone significantly improved consistency by over 8%. The addition of material (M) further enhanced semantic richness, while the inclusion of risk-level indicators (R) contributed the most to domain-specific expressiveness and task relevance. This analysis confirms the effectiveness of the proposed semantic cue lexicon and highlights the dimension-wise contribution of each cue to language generation quality.

**Figure 10:**
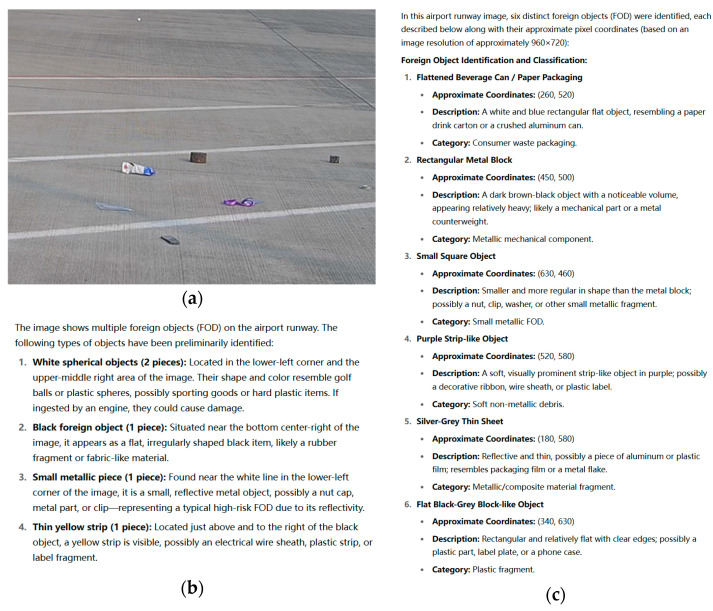
Comparison of responses before and after introducing spatial positioning and knowledge guidance. (**a**) Input FOD image; (**b**) semantic description before knowledge guidance; (**c**) semantic description after knowledge guidance.

Figure 10 illustrates the comparison of generated responses before and after the integration of spatial location and knowledge guidance. These findings strongly validate the effectiveness of the structured prompt strategy in enhancing language generation quality, particularly in the normative use of terminology, completeness of scene semantics, and explicit expression of risk information. The proposed structured prompt engineering framework significantly improves the accuracy and semantic consistency of foreign object descriptions.

#### 4.2.4. Runtime Breakdown and Deployment Considerations

To better understand the practical deployment feasibility of the proposed YOLO-SAM-GPT framework, we conducted a detailed runtime analysis, breaking down the computational cost of each major module. The experiments were conducted on a workstation with an Intel Xeon Gold 6226R CPU, 128 GB RAM, and an NVIDIA RTX 3090 GPU. Each timing result represents the average per image inference time over 500 test samples.

As shown in Table 12, the YOLOv7-X detector operates efficiently, requiring only 40.2 ms per image for inference. The SAM segmentation module, prompted with bounding boxes enlarged by 150%, consumes approximately 142.5 ms per image, as it generates high-resolution instance masks for multiple FOD regions. The structured prompt construction stage, which includes spatial encoding and semantic cue fusion, introduces a relatively minor overhead of 23.5 ms. The most time-intensive component is the large language model, which takes about 178.6 ms on average to produce a complete environmental description per image.

The total end-to-end latency sums to 384.8 ms per image, equivalent to approximately 2.6 frames per second. While this latency does not satisfy strict real-time requirements for high-frame-rate video applications, it remains acceptable for semi-real-time deployment, such as scheduled inspections or edge-assisted asynchronous surveillance systems. These use cases prioritize semantic interpretability and detection robustness over ultra-low latency.

#### 4.2.5. Robustness and Generalization Under Complex Conditions

**Figure 11:**
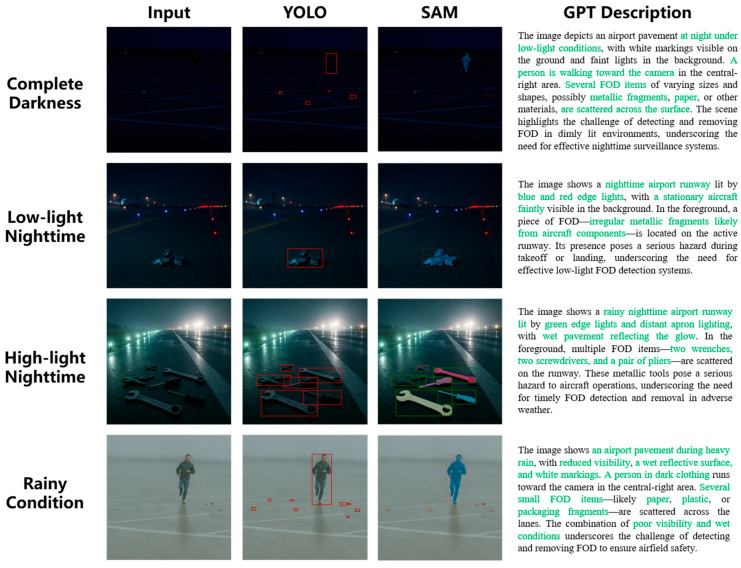
Visualization of FOD detection and spatial localization under complex conditions.

To evaluate the robustness and generalization capability of the proposed YOLO-SAM-GPT framework in challenging airport environments, we conducted additional qualitative experiments under four representative complex conditions: complete darkness, low-light nighttime, high-glare nighttime, and rainy weather. As shown in Figure 11, the model exhibits adaptability across these diverse scenarios.

In complete darkness, where the background is nearly invisible to human observers, the enhanced YOLO module successfully detects high-contrast FOD using learned structural and contextual priors; however, extremely low illumination occasionally leads to missed detections of small or low-reflectivity debris. In low-light nighttime scenes with minimal ambient illumination, the system maintains reliable detection and segmentation performance, leveraging texture-level cues. Under strong artificial lighting at night—commonly found near aprons or hangars—the model effectively handles intense glare and reflections, correctly localizing and describing small metallic debris. In rainy conditions, despite the SAM segmentation remaining robust for most objects, water-induced blur, reduced contrast, and motion artifacts occasionally cause false positives or inaccurate attribute descriptions. These examples highlight both the strengths and current limitations of the framework, confirming its deployment potential in low-visibility and weather-degraded environments while indicating areas for further improvement.

Future work should focus on improving robustness against detection errors through iterative refinement, feedback-based detection–segmentation loops, and complementary region proposals. Incorporating multi-modal inputs may mitigate failures in complete darkness or severe weather, while model compression and lightweight design could enable deployment in low-resource airport environments and on edge devices.

## 5. Conclusions

This paper presents an integrated framework for real-time detection and a semantic description of FOD on airport pavements. The proposed method addresses three critical challenges in this domain: the difficulty of detecting small objects under complex environments, the need for recognizing previously unseen FOD categories, and the demand for structured, interpretable scene-level understanding. The system combines a multi-scale feature enhancement detector based on YOLOv7-X, a SAM-guided segmentation pipeline for open-set FOD localization, and a structured prompt engineering mechanism for LLM-based semantic description. Extensive experiments on real-world datasets demonstrate significant improvements in detection accuracy, segmentation precision, and language controllability.

The main conclusions of this study are summarized as follows:

1. The proposed feature enhancement techniques effectively improve small-object detection performance. By incorporating Coordinate Attention, Space-to-Depth Convolution, and Gaussian similarity-based IoU assignment, the optimized YOLOv7-X model achieves an AP of 90.5%, with a 4.9% improvement over the baseline. Notably, the APs for small objects increased by 4.5–6.0%, demonstrating the effectiveness of preserving spatial detail and enhancing anchor assignment.
2. The cascaded YOLO-SAM segmentation framework improves mask precision and enables generalization to unannotated FOD instances. By analyzing different prompt strategies, it was found that moderate enlargement of YOLO-generated bounding boxes (150%) as prompts significantly improves SAM segmentation results. The optimal configuration achieves an MPA of 96.88, MIoU of 0.986, and F1 Score of 0.988, enabling reliable delineation of unknown foreign objects in diverse scenes.
3. The structured prompt engineering module improves the quality and consistency of GPT-generated FOD descriptions. By combining spatial encoding and a semantic cue lexicon, the system boosts description accuracy from 76.4% to 91.3%, and improves prompt consistency by 28.4%. This demonstrates that integrating expert knowledge and spatial priors into prompt templates significantly enhances LLM controllability and output interpretability.

## Figures and Tables

**Table 1:** Comparison of detection results among YOLO series models.

Model	AP (%)	AP_s_ (%)	AP_m_ (%)	AP_l_ (%)	Model Size (MB)	FPS
YOLOv5-X	82.1	73.1	84.3	87.7	86.7	46
YOLOX-X	84.2	76.5	85.1	90.7	99.1	27
YOLOv7	84.3	77.0	85.3	91.0	36.9	134
YOLOv7-X	85.5	77.6	87.6	91.3	71.3	90
YOLOv8-X	85.1	77.3	87.0	91.1	96.5	58
Deformable-DETR	82.8	74.0	85.0	90.5	160.4	12

**Table 2:** Impact of attention mechanism on model performance.

Attention Module	AP (%)	AP_s_ (%)	AP_m_ (%)	AP_l_ (%)
Baseline	85.5	77.6	87.0	91.3
SE	84.9	75.9	86.8	91.5
CBAM	86.9	77.9	89.1	93.6
CA (Proposed)	87.4	78.5	89.6	94.0

**Table 3:** Impact of the SPD-Conv module on model accuracy.

SPD-Conv Applied	AP (%)	AP_s_ (%)	AP_m_ (%)	AP_l_ (%)
✗ (Baseline)	85.5	77.6	87.0	91.3
✓ (Enabled)	88.3	83.6	89.4	91.6

**Table 4:** Impact of the spatial-to-depth transformation module on model accuracy.

Detector	GS-IoU	AP (%)	AP_s_ (%)	AP_m_ (%)	AP_l_ (%)
Faster R-CNN	✗	80.5	71.6	81.2	85.4
	✓	84.8	75.5	85.6	90.0
YOLOv5-X	✗	82.1	73.1	84.3	87.7
	✓	86.4	76.9	88.7	92.3
YOLOv7-X	✗	85.5	77.6	87.6	91.3
	✓	90.5	82.1	92.7	96.6

**Table 5:** Accuracy comparison of different IoU calculation methods on YOLOv7-X.

Method of IoU	AP (%)	AP_s_ (%)	AP_m_ (%)	AP_l_ (%)
IoU	72.4	67.3	75.3	81.4
CIoU	85.5	77.6	87.6	91.3
DIoU	70.1	64.8	74.5	85.6
GSIoU	88.5	80.6	89.5	92.6

**Table 6:** Computational cost comparison between CIoU and GSIoU.

Metric	CIoU	GSIoU	Relative Change
Training time per epoch (min)	68.5	64.7	+5.5%
Inference latency (ms)	8.74	8.89	+1.7%
mAP@0.5 (small objects)	77.6%	80.6%	+3.9%

**Table 7:** Evaluation metrics of SAM segmentation under different combinations of center point offset rates and anchor box scaling rates.

Center Point Offset	Anchor Box Scaling	MPA (%)	MIoU	F1 Score
0%	100%	96.85	0.982	0.983
0%	50%	36.85	0.583	0.602
0%	150%	96.88	0.986	0.988
20%	100%	85.24	0.875	0.884
20%	50%	68.36	0.763	0.778
20%	150%	88.35	0.912	0.932
40%	100%	76.58	0.823	0.842
40%	50%	48.35	0.658	0.702
40%	150%	82.63	0.852	0.865
80%	50%	23.75	0.435	0.495
80%	100%	59.34	0.714	0.792
80%	200%	81.84	0.832	0.854

**Table 8:** Performance comparison of basic environmental description generation models.

Model Name	Description Accuracy ↑	Detail Richness ↑	Language Fluency ↑	Prompt Controllability ↑	Average Inference Time ↓
BLIP-2	90.5%	88.3%	92.0%	91.5%	1.2 s/image
GIT	88.9%	89.6%	94.2%	89.7%	1.5 s/image
MiniGPT-4	82.7%	76.4%	87.5%	70.1%	0.9 s/image
PaLI	85.3%	83.0%	90.4%	75.2%	1.4 s/image

**Table 9:** Basic environmental description of FOD images.

Images	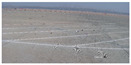	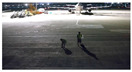	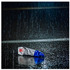	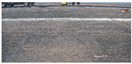
BLIP-2	The image shows an airport taxiway area with anti-slip concrete ground, foggy natural lighting with low visibility, and surrounding taxiway markings and light boxes.	The image depicts an airport apron with concrete ground, brightly illuminated by high-intensity lighting at night, surrounded by passenger stairs, jet bridges, and parked aircraft.	The image shows a rainy airport runway or apron with wet concrete or asphalt ground reflecting strong light glare, with no obvious surrounding facilities.	The image presents a sunny airport runway with dry cement or asphalt ground, sufficient illumination, located near the runway edge, surrounded by work vehicles, personnel, and some equipment.
GIT	Concrete ground under daytime natural lighting with some fog, located in an airport runway area, surrounded by sparse signage facilities; debris, such as plastic bottles and metal cans, scattered on the ground.	Concrete ground illuminated by artificial light at night, located in an airport apron area, surrounded by aircraft, boarding stairs, and ground service vehicles.	Asphalt-like ground under a rainy night with low light, unclear spatial location, and no obvious surrounding facilities.	Concrete ground under natural lighting with slightly overcast weather, located at Xuzhou Guanyin International Airport runway area, surrounded by work vehicles and fences, with small amounts of scattered debris.
MiniGPT-4	Airport runway area under cloudy lighting with concrete ground containing foreign objects, such as plastic bottles and paper scraps, with distant surrounding facilities.	Dark gray concrete airport apron brightly lit at night, surrounded by aircraft, boarding stairs, and vehicles.	Wet asphalt road under dim lighting in a city with dense rain, with no obvious surrounding facilities.	A wide concrete or asphalt-paved airport runway area under overcast natural light, located within Xuzhou Guanyin International Airport, surrounded by staff, engineering vehicles, ground markings, work equipment, and distant fences.
PaLI	The image shows an airport cement runway under cloudy lighting with scattered colored plastic debris as foreign objects, sparse surrounding facilities, and blurred tree outlines in the distance.	The image depicts an airport apron cement ground at night under artificial lighting, with aircraft, boarding stairs, and ground service vehicles; overall illumination is bright and evenly distributed.	The image shows a rainy night asphalt road with reflective wet ground, dim lighting, and dense rain visible, with unclear spatial location and surrounding facilities.	The image presents a gray cement airport runway under soft daytime lighting, with a warning vehicle parked on the left, visible cardboard boxes on the right, and multiple workers wearing reflective vests conducting inspections in the central area.

**Table 10:** Performance comparison of structured prompt.

Experimental Group	Prompt Type	Semantic Cue Lexicon	Spatial Location	Description Accuracy ↑	Prompt Consistency ↑
A	Generic Natural Language	✗	✗	76.4%	61.2%
B	+ Spatial Location	✗	✓	83.7%	74.8%
C	+ Semantic Cues + Spatial Location	✓	✓	91.3%	89.6%

**Table 11:** Dimension-wise contribution of semantic cues to prompt performance.

Prompt Group	Spatial (S)	Material (M)	Risk Level (R)	Description Accuracy ↑	Prompt Consistency ↑
A (Base)	✗	✗	✗	76.4%	61.2%
B (Base + S)	✓	✗	✗	82.3%	69.6%
C (Base + S + M)	✓	✓	✗	87.5%	79.4%
D (Full)	✓	✓	✓	91.3%	89.6%

**Table 12:** Average inference time breakdown of major modules.

Module	Description	Average Inference Time (ms/image)
YOLOv7-X Detection	Bounding box generation	40.2
SAM Segmentation	Instance segmentation via bounding box prompting	142.5
Prompt Construction	Structured template generation and keyword fusion	23.5
LLM Description Generation	Image-to-text via BLIP-2	178.6
Total	—	384.8

## Data Availability

Please contact the corresponding author to request access to the data mentioned in this article, but note that it cannot be used for commercial activities.

## Appendix A

### Appendix A.1. Structured Prompt Templates

This section provides the exact templates used in the structured prompt system for generating descriptions of detected foreign objects on airport pavements. The templates are designed to incorporate information related to spatial location, material properties, environmental conditions, and operational risks.

The following template serves as the foundation for generating semantic descriptions of FOD instances.

Prompt Format: “Please describe the foreign object number [ID], located at [spatial location], with ground material [material type], under lighting conditions [lighting type]. Surrounding facilities include [facilities]. The object is [risk level] for operational safety.”

Example Prompt: “Please describe the foreign object number 2, located at the eastern edge of the runway, with ground material asphalt, under lighting conditions of strong glare. Surrounding facilities include drainage ditches. The object is high risk for operational safety.”

### Appendix A.2. Semantic Lexicon

Foreign Object Categories

These refer to the type of material or object detected on the runway or taxiway. Common categories include the following: metal, plastic, textile, wood, paper, glass, rubber, fabric, cloth, concrete debris, screws, nuts, tape, packaging, etc.

Spatial Location Categories

These describe where the object is located within the airport area, including the following: runway centerline, taxiway edge, apron, runway edge, near aircraft, near runway markers, near signage, along flight path, etc.

Lighting Conditions

These reflect the lighting environment in the image, which can affect object visibility and appearance, including the following: direct sunlight, low-light nighttime, strong glare, backlit, dim, artificial lighting, foggy conditions, rainy conditions, overcast lighting, etc.

Ground Material Types

These reflect the lighting environment in the image, which can affect object visibility and appearance, including the following: concrete, asphalt, grass, water-covered, dirt, snow-covered, ice, pavement cracks, etc.

Surrounding Facilities

These describe any infrastructure or facilities nearby that can help provide context for the object, including the following: boundary lighting strips, drainage ditches, fences, work vehicles, runway markings, ground service vehicles, taxiway signage, airplane docking areas, etc.

Risk Levels

These indicate the operational safety risk associated with the foreign object, including the following: high risk, medium risk, low risk, non-critical, immediate removal required, potential runway hazard, obstacle near takeoff zone, etc.

### Appendix A.3. Usage of Structured Prompt System in This Paper

In the methodology section of this paper, the generated prompts are fed into the GPT-based model to produce the corresponding descriptions for detected FOD. This system ensures that the generated textual output is aligned with the spatial, contextual, and risk-related aspects of the detection scenario.
